# Surgical repair of isolated total anomalous pulmonary venous connection is linked to favourable outcomes.

**DOI:** 10.1186/1749-8090-10-S1-A324

**Published:** 2015-12-16

**Authors:** Kostas Kostopanagiotou, Andrew Chatzis, Costas Contrafouris, Fotios Mitropoulos, Prodromos Azariades

**Affiliations:** 1 Department of Pediatric & Congenital Cardiac Surgery, Onassis Cardiac Surgery Centre, Athens 17674, Greece

## Background/Introduction

The operative mortality for total anomalous pulmonary venous connection repair is considered high when coexists with other congenital cardiac lesions. The isolated form however warrants excellent surgical outcomes.

## Aims/Objectives

We analysed our series to test the validity of the above statement.

## Method

From July 2007-April 2015, 18 consecutive patients,10 males and 8 females (55/45%), median age 33 months with isolated total anomalous pulmonary venous connection were operated in our department. Of these 8 (44.4%) were of the supracardiac type, 4 (22.2%) cardiac, 5 (27.8%) of the infracardiac type and one mixed case (5.6%). Perinatal respiratory distress was the prominent clinical symptom (61%). Mean cardiopulmonary by-pass time/cross-clamp time was 128/69 minutes. In all cases except the cardiac type repair was performed with direct anastomosis of the connecting vein to the left atrium and atrial septal defects were closed with autologous pericardium.

## Results

There was one early mortality (5.5%) related to marked pulmonary hypertension and one case of postoperative chylothorax managed conservatively. Median intensive care unit stay was 7 and length of hospital stay 15 days respectively. Mean follow-up was 22 months (range 1-70) with trivial tricuspid regurgitation in 5 patients (33.3%) and moderate in one patient (6.6%). During this period no pulmonary venous stenosis was observed. No reoperation was necessary and no late deaths.

## Discussion/Conclusion

Isolated total anomalous pulmonary venous connection is usually diagnosed after birth unlike complex forms where their pathology is conspicuous. The isolated form can be safely operated early with excellent surgical outcomes.

